# Linear programming model can explain respiration of fermentation products

**DOI:** 10.1371/journal.pone.0191803

**Published:** 2018-02-07

**Authors:** Philip Möller, Xiaochen Liu, Stefan Schuster, Daniel Boley

**Affiliations:** 1 Dept. of Bioinformatics, Friedrich-Schiller-Universität, Jena, Germany; 2 Dept. of Computer Science and Eng., University of Minnesota, Minneapolis, MN, United States of America; Friedrich-Alexander-Universitat Erlangen-Nurnberg, GERMANY

## Abstract

Many differentiated cells rely primarily on mitochondrial oxidative phosphorylation for generating energy in the form of ATP needed for cellular metabolism. In contrast most tumor cells instead rely on aerobic glycolysis leading to lactate to about the same extent as on respiration. Warburg found that cancer cells to support oxidative phosphorylation, tend to ferment glucose or other energy source into lactate even in the presence of sufficient oxygen, which is an inefficient way to generate ATP. This effect also occurs in striated muscle cells, activated lymphocytes and microglia, endothelial cells and several mammalian cell types, a phenomenon termed the “Warburg effect”. The effect is paradoxical at first glance because the ATP production rate of aerobic glycolysis is much slower than that of respiration and the energy demands are better to be met by pure oxidative phosphorylation. We tackle this question by building a minimal model including three combined reactions. The new aspect in extension to earlier models is that we take into account the possible uptake and oxidation of the fermentation products. We examine the case where the cell can allocate protein on several enzymes in a varying distribution and model this by a linear programming problem in which the objective is to maximize the ATP production rate under different combinations of constraints on enzymes. Depending on the cost of reactions and limitation of the substrates, this leads to pure respiration, pure fermentation, and a mixture of respiration and fermentation. The model predicts that fermentation products are only oxidized when glucose is scarce or its uptake is severely limited.

## Introduction

Many living cells use different biochemical pathways like fermentation and respiration in their core energy metabolism, depending on the availability of and competition for the necessary substrates. Examples are provided by baker’s yeast as well as striated muscle and tumor cells in humans. Fermentation, by which sugar is converted to lactate, ethanol or the like, is a low-yield process because only two moles of the cell’s energy currency, ATP are produced per mole of glucose. In contrast, respiration, which requires oxygen and can use glucose or a similar substrate as well, has a much higher yield. For tumor cells, the fact that fermentation is used in addition to respiration was first discovered by Otto H. Warburg in the 1920’s [1]. He was hypothesizing that it resulted from impaired function of mitochondria, leading to tumor growth [1, 2]. Accordingly, that behavior is called “Warburg effect”, even for other cell types [3–6].

Previously, several models based on linear programming with some specific constraints to explain the higher ATP production rate in the Warburg effect during the respiro-fermentation process in many of these cell types were established [4–8]. Our model [6, 7] is the smallest of these, still describing the essential features, and can be considered as a minimalist model. We considered that the cell can allocate protein on several enzymes in a varying distribution. Depending on side conditions and on protein costs, this led to pure respiration, pure glycolysis, and respiro-fermentation as a mixed flux distribution. Other models of resource allocation in living cells have been proposed and analyzed in [9–12].

Our previous model did not cover the case where respiration uses, as a substrate, a low-energy metabolite that is a product of fermentation, such as lactate, acetate or ethanol. However, this is a wide-spread phenomenon. Respiration of fermentation products occurs in two different ways. In some cell types, the two pathways are used consecutively in time. This dynamic phenomenon was first described by Jacques Monod in 1949 for *Escherichia coli* [13] and was termed “diauxie”. Today the “diauxic shift” is known to occur in *Saccharomyces cerevisiae* as well [14].

First sugar is used as the preferred substrate to grow as fast as possible. When the metabolite is depleted, the organism switches to another energy source like lactate or acetate, which in many cases were excreted by the same organism before. A related process is the respiration of fatty acids. In a first phase, sugar is transformed, via acetyl-CoA, into fatty acids to store energy and carbons. When needed, these stores are converted back to acetyl-CoA and fed into the respiratory pathway.

The second possibility is that the low-yield and high-yield pathways are employed by different cells which cooperate in a division of labour called sequential cross-feeding. One cell excretes lactate (or a similar metabolite), while the other takes it up. For example, different strains of *E. coli* can coexist through cross-feeding [15]. This flexibility of using carbon sources is not only limited to bacteria or yeasts but was also shown to occur in higher organisms. In the human organism there are at least two examples of pairs of “sequential cross-feeding” (although this term is mostly used in microbiology). One example is the cooperation between astrocytes and neurons [16]. The former mainly degrade glucose to lactate (partially they respire in addition), while neurons mainly convert lactate to CO2 and water by respiration. Moreover, neurons can take up, from the blood, lactate that was produced by other tissues. For instance, when lactate is produced by muscle cells under high workload, it is available for the brain, which then stops producing lactate itself and consumes it instead [16].

In this study, we extend our earlier simple linear programming (LP) model [6, 7] for the maximal production rate encompassing the trade-off between respiration and fermentation by allowing the uptake and oxidation of low-energy metabolites. To that end, we declare the reaction transforming pyruvate into the fermentation product as reversible, while it was declared irreversible in the earlier model. Again, we explore the parameter space to find all possible solutions to the corresponding linear program and interpret the solutions in biological terms.

**Notation:** vectors are represented by lower case bold letters, like **v** = (*v*_1_; *v*_2_; *v*_3_) (where “;” means vertical concatenation *à la* Matlab). This notation normally represents a column vector. The row vector with the same entries is its transpose: **v**^*T*^.

## Materials and methods

**Fig 1:**
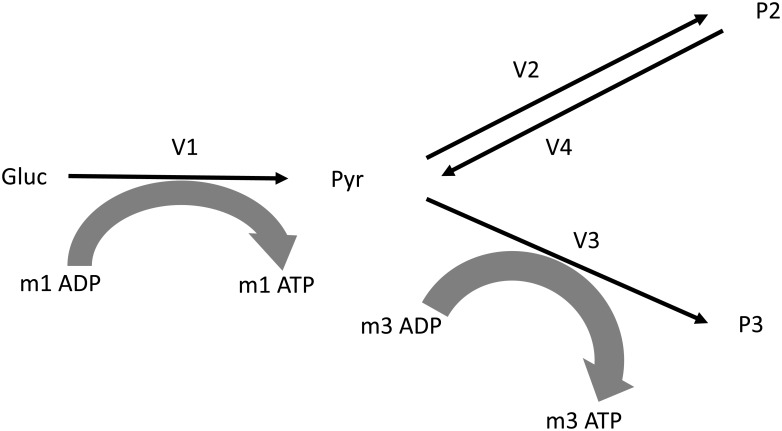
Minimal reaction scheme for analyzing reversed fermentation reaction in Warburg effect. Gluc, glucose; Pyr, pyruvate; *P*_2_, fermentation product including lactate and ethanol; *P*_3_, respiration products including carbon dioxide and water; *v*_*i*_, reaction rates; *m*_*i*_, stoichiometric coeficients of ATP. *m*_1_ = 2, *m*_3_ = 30.

Guided by the principle of Ockham’s razor that a simplest model capable of explaining a complicated process is preferred, we establish a minimal model that attempts to fully explain the Warburg effect without overlooking the scientific nature built in the phenomenon. Our model consists of three main reactions, each of which represents a complex pathway. The first reaction is glycolysis, the conversion of glucose into pyruvate. The second reaction is the fermentation of pyruvate into lactate or alcohol by an anaerobic process. The third reaction is the combination of tricarboxylic acid (TCA) cycle and oxidative phosphorylation, the aerobic process to extract and store energy in the form of ATP and waste products “P_3_”. In this paper, we consider a fourth reaction capable of consuming lactate to produce pyruvate. This is almost equivalent to allowing the second reaction (fermentation) to be reversible, but the enzymes allocated to the reverse reaction may imply different costs compared to the forward reaction. To simplify the terminology used in this paper, we use the term “respiration” to refer specifically to the respiration (TCA cycle plus oxidative phosphorylation) of pyruvate to *P*_3_, the term “fermentation” to refer to the fermentation of pyruvate to *P*_2_ (e.g., lactate), and the term “reverse fermentation” to refer to the reaction converting lactate back to pyruvate. This is in contrast to the common practice to use respiration and fermentation to mean the entire pathway from glucose to the respective final product. The result is the model shown in Fig 1.

In our minimal model, one mole of glucose is consumed by reaction 1 (denoted by *v*_1_) to produce two moles of pyruvate and *m*_1_ moles of ATP. We consider the pyruvate an internal metabolite and all other substrates external metabolites having fixed concentrations. Fermentation is modelled by the pathway leading from pyruvate (product of *v*_1_) to *P*_2_—for simplicity, we refer to lactate only. Part of the fermentation products can be reused to regenerate pyruvate, which can be further used by oxidative phosphorylation. In these cases, the flux of the second reactions *v*_2_ will be negative. In a few cases, it is known that *v*_2_ will also produce a few ATP (e.g., acetate fermentation by *Escherichia coli*, where we could set *m*_2_ = 1). But since this is rare in ATP production via fermentation, we ignore it in our model by using *m*_2_ = 0. Respiration leads to P_3_ producing *m*_1_ moles of ATP during the initial glycolysis and *m*_3_ moles of ATP during the final aerobic conversion of pyruvate to waste product P_3_. The typical values *m*_1_ = 2 and *m*_3_ = 30 are used [17]. We impose the simplest possible objective for a cell, namely to produce ATP as fast as possible. Our simple model can be augmented in the future to include neglected effects such as consumption/production of NADH, or ATP in the fermentation reaction, or variations in pathway costs. We count all rates and stoichiometric coefficients in terms of consumed moles of glucose.

To describe the Warburg effect, we phrase it as a linear programming optimization model. Our model mimics the real metabolic activities within the organism but is simple to understand and solve without loss of generality.

We introduce our model with unlimited substrate availablity first and then consider the model with limited substrate availability in a separate section. The enzyme concentration in the pathways of glycolysis and oxidative phosphorylation are regarded to be variable because they can change both during evolution and development of a given organism. Thus, re-allocation of protein between enzymes and pathways is allowed for.

We adopt a simple model for the use of internal resources (protein) within the cell. We say that one (arbitrary) unit of glycolysis (*v*_1_) uses *α*_1_ (arbitrary) units of internal resources, one unit of respiration (*v*_3_) uses *α*_3_ units of internal resources, and one unit of fermentation (*v*_2_) uses *α*_2_ units in the forward direction (*v*_2_ > 0) and ${\hat{\alpha }}_{2}$ units when in the reverse direction (*v*_2_ < 0). The total use of internal resources is limited by the availability of amino acids to form the respective enzymes. This is modelled mathematically by imposing an upper limit Γ on the weighted sum of the reaction fluxes. The quantities *α*_1_, *α*_2_, *α*_3_, Γ measuring the internal resources are in abstract units of “enzyme load”. Their importance lies in their mutually relative values. This gives rise to two alternative constraints

$$\begin{array}{c} \begin{array}{cc} \alpha_{1}v_{1}+\alpha_{2}v_{2}+\alpha_{3}v_{3}\leq \Gamma & \left(\text{cost}\;\text{constraint}\;\text{for}\;\text{forward}\;\text{reaction,}\;v_{2}\geq 0\right)\\ \alpha_{1}v_{1}-{\hat{\alpha }}_{2}v_{2}+\alpha_{3}v_{3}\leq \Gamma & (\text{cost}\;\text{constraint}\;\text{for}\;\text{reverse}\;\text{reaction,}\;v_{2}\leq 0). \end{array} \end{array}$$

These appear as constraints (c) & (d), respectively, in the optimization model (1) given below. We assume both parameters *α*_2_ and ${\hat{\alpha }}_{2}$ are positive (or at least non-negative). In this case, the first constraint (c) is tighter than the second constraint (d) for positive *v*_2_ while (d) is tighter for negative *v*_2_. Hence, even though only one constraint is relevant for any particular value of *v*_2_, we can include both constraints into a single linear programming model. In the second constraint (d), the negative sign in front of the “${\hat{\alpha }}_{2}$” accounts for the fact that in this formula *v*_2_ is itself negative, but gives rise to a positive cost.

### Model—Unlimited substrate

We analyze the following optimization problem:

$$\begin{array}{c} \begin{array}{cccc} \max & \left(\text{a}\right) & \phi \left(v\right)=m^{T}v=m_{1}v_{1}+m_{3}v_{3} & \left(\text{ATP}\;\text{production}\right)\\ \text{s}.\text{t}. & \left(\text{b}\right) & v_{1}-v_{2}-v_{3}=0 & \left(\text{mass}\;\text{balance}\right)\\ & \left(\text{c}\right) & \alpha_{1}v_{1}+\alpha_{2}v_{2}+\alpha_{3}v_{3}\leq \Gamma & \left(\text{cost}\;\text{constraint}\;\text{for}\;\text{forward}\;\text{reaction}\right)\\ & \left(\text{d}\right) & \alpha_{1}v_{1}-{\hat{\alpha }}_{2}v_{2}+\alpha_{3}v_{3}\leq \Gamma & \left(\text{cost}\;\text{constraint}\;\text{for}\;\text{reverse}\;\text{reaction}\right)\\ & \left(\text{e}\right) & v_{1}\geq 0 & \left(\text{irreversibility}\;\text{of}\;v_{1}\right)\\ & \left(\text{f}\right) & v_{3}\geq 0 & \left(\text{irreversibility}\;\text{of}\;v_{3}\right) \end{array} \end{array}$$ (1)

As the metabolic system is considered to be at steady state, we use (b) to eliminate *v*_1_, so that the entire model is expressed in terms of just two variables *v*_2_ and *v*_3_.

$$\begin{array}{c} \begin{array}{cccc} \max & \left(\text{a}\right) & \phi \left(v\right)=m^{T}v=m_{1}v_{2}+\left(m_{1}+m_{3}\right)v_{3} & \;(\text{ATP}\;\text{production}\;)\\ \text{s}.\text{t}. & \left(\text{c}\right) & \left(\alpha_{1}+\alpha_{2}\right)v_{2}+\left(\alpha_{1}+\alpha_{3}\right)v_{3}\leq \Gamma & \left(\text{cost}\;\text{constraint}\;\text{for}\;\text{forward}\;\text{reaction}\right)\\ & \left(\text{d}\right) & \left(\alpha_{1}-{\hat{\alpha }}_{2}\right)v_{2}+\left(\alpha_{1}+\alpha_{3}\right)v_{3}\leq \Gamma & \left(\text{cost}\;\text{constraint}\;\text{for}\;\text{reverse}\;\text{reaction}\right)\\ & \left(\text{e}\right) & v_{2}+v_{3}\geq 0 & (\text{irreversibility}\;\text{of}\;v_{1})\\ & \left(\text{f}\right) & v_{3}\geq 0 & (\text{irreversibility}\;\text{of}\;v_{3}) \end{array} \end{array}$$ (2)

The objective function is to maximize the ATP production rate given by a linear combination of rates with the stoichiometric coefficients of ATP as weighting factors. The constraints (c) and (d) reflect the organism’s resource limit in creating enzymes for the forward fermentation reactions and reverse reactions, respectively. Note that the maximal velocities of enzymes are given by the products of enzyme concentrations and turnover numbers [6]. The coefficients *α*_*i*_ express the different turnover numbers as well as the different synthesis costs of enzymes, which are largely determined by the molar masses of the enzymes and the different synthesis costs of amino acids [4, 5, 9, 18, 19]. Written in terms of enzyme concentrations, such a side constraint has been used in metabolic modeling earlier [20, 21]. Furthermore, relations (c) and (d) can reflect macromolecular crowding [4].

Moreover, we can assume that reactions 1 and 3 are irreversible because they include at least one irreversible partial reaction, hence we include constraints (e) and (f) in our model. For example, the hexokinase and phosphofructokinase reactions involved in glycolysis are irreversible. The second reaction may be reversible (e.g. lactate dehydrogenase or alanine aminotransferase) such as neurons taking up lactate and respiring it for energy source [22].

**Fig 2:**
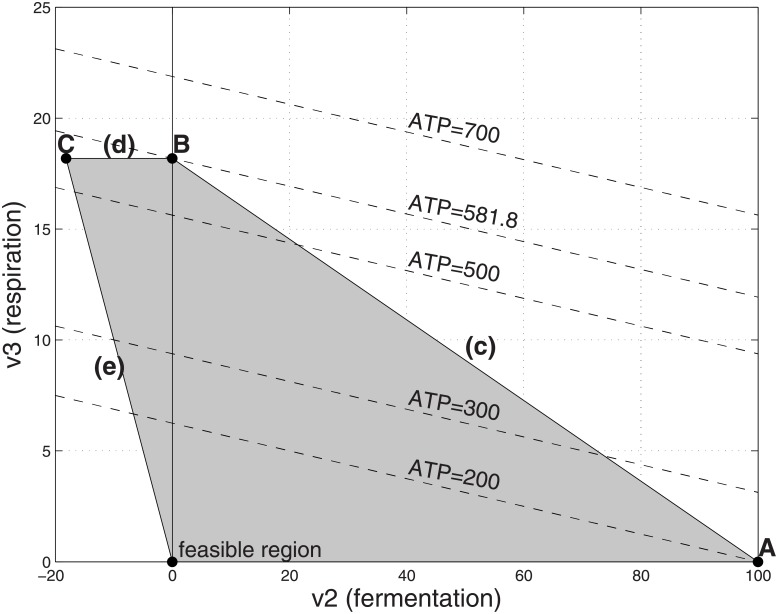
Feasible flux region with low cost respiration and unlimited substrate. *α*_1_ = 1, *α*_2_ = 1, *α*_3_ = 10, Γ = 200, ${\hat{\alpha }}_{2}=1$ and V_1_ = ∞ (entire shaded region). Lower case letters refer to constraints. Also shown are the level contours for the objective function ATP. The optimal solution is at point B in which (*v*_2_, *v*_3_) = (0, 18.18), corresponding to *v*_1_ = 18.18, ATP = 581.8 and a yield ratio of ATP/*v*_1_ = 32.

**Fig 3:**
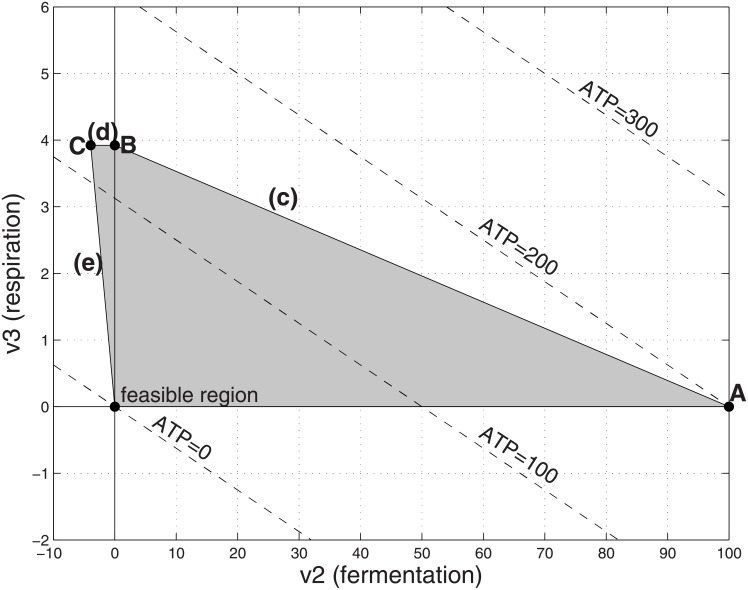
Feasible flux region with high cost respiration and unlimited substrate. Parameters as in Fig 2 except *α*_3_ = 50. The optimal solution is at point A, in which (*v*_2_, *v*_3_) = (100, 0), corresponding to *v*_1_ = 100, ATP = 200 and a yield ratio of ATP/*v*_1_ = 2.

**Fig 4:**
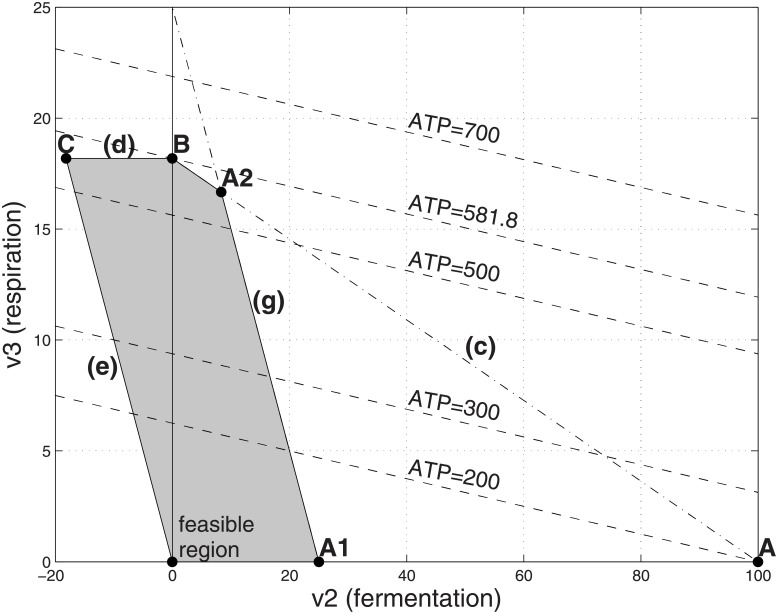
Feasible flux region with low cost respiration and limited substrate. Parameters as in Fig 2 except that substrate uptake *v*_1_ is capped by a big value (V_1_ = 25). The optimal solution is at point B in which (*v*_2_, *v*_3_) = (0, 18.18), corresponding to *v*_1_ = 18.18, ATP = 581.8 and a yield ratio of ATP/*v*_1_ = 32.

**Fig 5:**
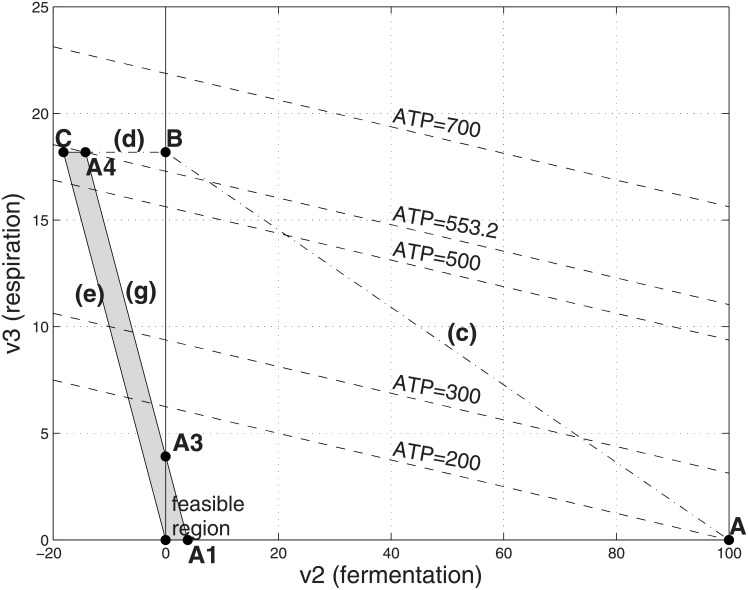
Feasible flux region with low cost respiration and very tight limit on substrate. Parameters as in Fig 2 except that substrate uptake *v*_1_ is capped by a small value (V_1_ = 3.92). The optimal solution is at point A4 (mixture of respiro-fermentation and reverse fermentation) if the fermentation is reversible, in which (*v*_2_, *v*_3_) = (−14.26, 18.18), corresponding to *v*_1_ = 3.92, ATP = 553.24 and a yield ratio of ATP/*v*_1_ = 141. A3 marks the optimal point if the fermentation were irreversible, in which (*v*_2_, *v*_3_) = (0, 3.92), corresponding to *v*_1_ = 3.92, ATP = 125.44 and a yield ratio of ATP/*v*_1_ = 32. The vertical line through the origin indicates the left boundary of the admissible region in the case of irreversible fermentation.

**Fig 6:**
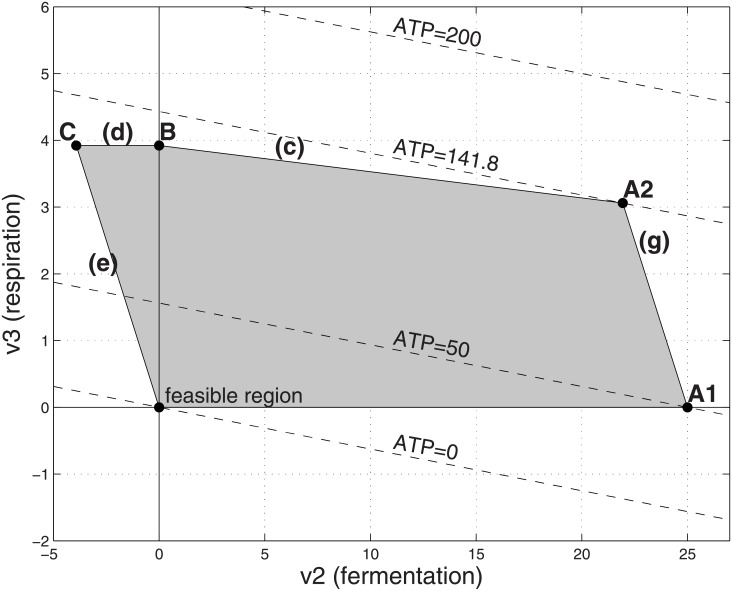
Feasible flux region with high cost respiration and limited substrate. Parameters as in Fig 2 except that *α*_3_ = 50 and the substrate uptake *v*_1_ is capped by a big value (V_1_ = 25). The optimal solution is at point A2, in which (*v*_2_, *v*_3_) = (21.94, 3.06), corresponding to *v*_1_ = 25, ATP = 141.8 and a yield ratio of ATP/*v*_1_ = 5.67.

**Fig 7:**
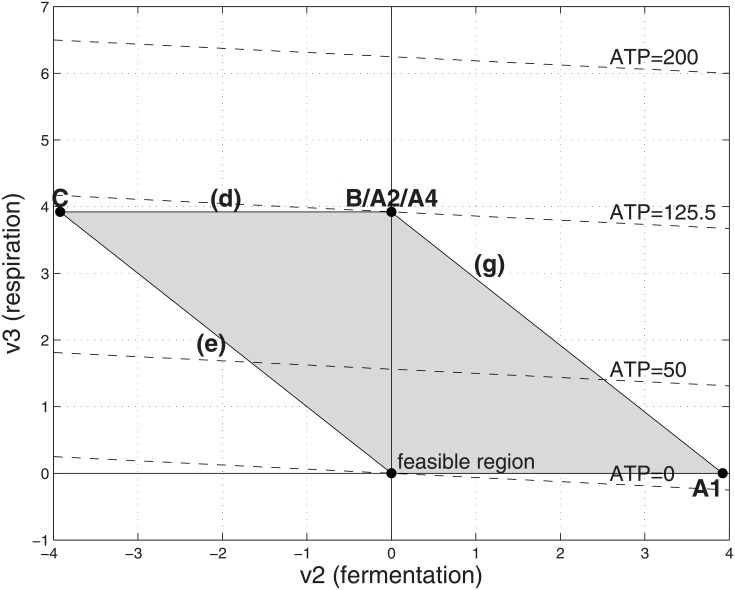
Feasible flux region with high cost respiration and very tight limit on substrate. Parameters as in Fig 2 except that *α*_3_ = 50 and the substrate uptake *v*_1_ is capped by a small value (V_1_ = 3.92). The optimal solution is at point A2/B/A4, in which (*v*_2_, *v*_3_) = (0, 3.92), corresponding to *v*_1_ = 3.92, ATP = 125.5 and a yield ratio of ATP/*v*_1_ = 32.02.

**Fig 8:**
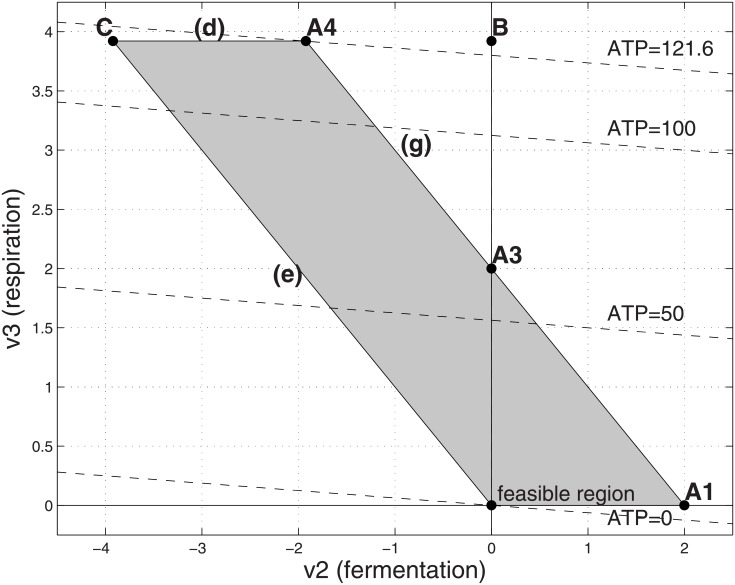
Feasible flux region with high cost respiration and even stricter limit on substrate. Parameters as in Fig 2 except that *α*_3_ = 50 and the substrate uptake *v*_1_ is capped by a even smaller amount (V_1_ = 2). The optimal solution is at point A4 if the fermentation is reversible, in which (*v*_2_, *v*_3_) = (−1.92, 3.92), corresponding to *v*_1_ = 2, ATP = 121.6 and a yield ratio of ATP/*v*_1_ = 60.8. It is at point A3 if the fermentation is irreversible, in which (*v*_2_, *v*_3_) = (0, 2), corresponding to *v*_1_ = 2, ATP, = 64 and a yield ratio of ATP/*v*_1_ = 32.

In the following, we assume that all the parameters *m*_1_, *m*_3_, *α*_1_, *α*_2_, *α*_3_, Γ, V_1_ are non-negative, where V_1_ is a limit on the uptake of available substrate to be considered in the next section. We also assume *α*_3_ > *α*_1_, *α*_3_ > *α*_2_, arising from the biochemical observation that respiration is strictly more costly than the last step of fermentation, because (among other reasons) the pathway is much longer and the enzymes of oxidative phosphorylation are located in a membrane [4, 8, 18]. System (2) is a linear program in two variables, which can be solved easily, and its feasible region can be analyzed graphically, as illustrated below in Figs 2–8.

It is well known (see e.g., [23]) that if there is a unique optimal solution, it must be at one of the vertices of the feasible region (which is a polyhedron). If the optimal solution is not unique, there will be at least two vertices that satisfy the optimum. So it suffices to look at the four vertices shown in Figs 2 and 3, marked A, B, C, and O. We show in Table 1 the general expression of these four points. Each vertex lies at the intersection of the two indicated binding constraints in (2) (i.e. where the two inequality constraints are satisfied with equality).

**Table 1:** Critical points for linear programming model with unlimited substrate.

Point	Binding Constraints	Values			
		*v*_1_	*v*_2_	*v*_3_	ATP value *ϕ*
O	(e)(f)	0	0	0	0
A	(c)(f)	$\frac{\Gamma }{\left(\alpha_{1}+\alpha_{2}\right)}$	$\frac{\Gamma }{\left(\alpha_{1}+\alpha_{2}\right)}$	0	$\frac{m_{1}\Gamma }{\left(\alpha_{1}+\alpha_{2}\right)}$
B	(c) & *v*_2_ ≥ 0	$\frac{\Gamma }{\left(\alpha_{1}+\alpha_{3}\right)}$	0	$\frac{\Gamma }{\left(\alpha_{1}+\alpha_{3}\right)}$	$\frac{(m_{1}+m_{3})\Gamma }{\left(\alpha_{1}+\alpha_{3}\right)}$
C	(d)(e)	0	$-\frac{\Gamma }{\left({\hat{\alpha }}_{2}+\alpha_{3}\right)}$	$\frac{\Gamma }{\left({\hat{\alpha }}_{2}+\alpha_{3}\right)}$	$\frac{m_{3}\Gamma }{\left({\hat{\alpha }}_{2}+\alpha_{3}\right)}$

With *v*_1_ eliminated, we analyse graphically the resulting LP (2) in the two variables *v*_2_, *v*_3_. Fig 2 shows the feasible region for (2) when respiration is low cost (e.g., *α*_3_ = 10) and the substrate resource is unlimited, modelled as imposing no limit on the the substrate uptake *v*_1_. The level contours for the objective function are shown in the figure, one of which is intersected with point B on the feasible region: the maximum feasible value for ATP occurs using pure respiration.

Next we consider the situation where the respiration is costly (e.g., *α*_3_ = 50) in Fig 3. The maximum feasible value for ATP production occurs when there is pure fermentation at point A. Comparing the ATP production values for points A and B in Table 1, one sees that the cross-over point between the situation where point A (pure fermentation) is optimal and point B (pure respiration) is optimal occurs when

$$\begin{array}{c} \frac{m_{1}\Gamma }{\alpha_{1}+\alpha_{2}}=\frac{(m_{1}+m_{3})\Gamma }{\alpha_{1}+\alpha_{3}} \end{array}$$

or as given in [6]

$$\begin{array}{c} \frac{m_{1}}{m_{1}+m_{3}}=\frac{\alpha_{1}+\alpha_{2}}{\alpha_{1}+\alpha_{3}} \end{array}$$ (3)

Pure fermentation (point A) is optimal when the left hand side of (3) is larger. With high-cost respiration subject to a limit on the total internal cost, the optimal operating point is obtained using the lowest cost process available (fermentation) even if more substrate must be consumed to achieve that level of ATP production. Using respiration to achieve that level of ATP production would exceed the internal resource limits.

### Model—Limited substrate

We now consider the case in which the uptake of the substrate such as glucose (P_1_) has a limit, represented by an additional constraint (g). This yields the following model, in which we have used the mass balance relation (b) to eliminate *v*_1_, as done to obtain (2) from (1):

$$\begin{array}{c} \begin{array}{cccc} \max & \left(\text{a}\right) & \phi \left(v\right)=m^{T}v=m_{1}v_{2}+\left(m_{1}+m_{3}\right)v_{3} & (\text{ATP}\;\text{production}\;\text{assuming}\;v_{1}=v_{2}+v_{3})\\ \text{s}.\text{t}. & \left(\text{c}\right) & \left(\alpha_{1}+\alpha_{2}\right)v_{2}+\left(\alpha_{1}+\alpha_{3}\right)v_{3}\leq \Gamma & \left(\text{cost}\;\text{constraint}\;\text{for}\;\text{forward}\;\text{reaction}\right)\\ & \left(\text{d}\right) & \left(\alpha_{1}-{\hat{\alpha }}_{2}\right)v_{2}+\left(\alpha_{1}+\alpha_{3}\right)v_{3}\leq \Gamma & \left(\text{cost}\;\text{constraint}\;\text{for}\;\text{reverse}\;\text{reaction}\right)\\ & \left(\text{e}\right) & v_{2}+v_{3}\geq 0 & (\text{irreversibility}\;\text{of}\;v_{1})\\ & \left(\text{f}\right) & v_{3}\geq 0 & (\text{irreversibility}\;\text{of}\;v_{3})\\ & \left(\text{g}\right) & v_{2}+v_{3}\leq V_{1} & (\text{substrate}\;\text{uptake}\;\text{limit}\;\text{on}\;v_{1}) \end{array} \end{array}$$ (4)

The optimal solution depends on the values of the parameters, but again must occur at a vertex. We list below the general expressions for the variables and the objective function at the various vertices. Analogously as above, we assume that ${\hat{\alpha }}_{2}$ is non-negative, and $\alpha_{3}>{\hat{\alpha }}_{2}$.

We first discuss the situation with high-cost respiration. The situations that can result from the model (4) can be divided into three main regimes, depending on whether the limit V_1_ on the available substrate P_1_ lies in the range $V_{1}\geq \frac{\Gamma }{\alpha_{1}+\alpha_{2}}$, $\frac{\Gamma }{\alpha_{1}+\alpha_{3}}<V_{1}<\frac{\Gamma }{\alpha_{1}+\alpha_{2}}$, or $V_{1}<\frac{\Gamma }{\alpha_{1}+\alpha_{3}}$. We illustrate the situations in Figs 3, 6, 7 and 8, using the numerical values *m*_1_ = 2, *m*_3_ = 30, *α*_1_ = 1, *α*_2_ = 1, *α*_3_ = 50, Γ = 200, ${\hat{\alpha }}_{2}=1$ [24].

If $V_{1}\geq \frac{\Gamma }{\alpha_{1}+\alpha_{2}}=100$, then the system behaves as if the substrate were unlimited, as described in the previous section. If $3.92=\frac{\Gamma }{\alpha_{1}+\alpha_{3}}\leq V_{1}<\frac{\Gamma }{\alpha_{1}+\alpha_{2}}=100$ then the optimal operating point corresponds to mixed fermentation and respiration, marked A2 in Fig 6. Here ATP production is maximized by using a mix of respiration and fermentation. There are insufficient internal resources to process the entire amount of substrate via pure costly respiration, but if the entire substrate were processed by pure fermentation, the organism would have left-over resources that are not used. Therefore the optimal operating point is a mix of the two pathways. It can be verified by algebraic manipulation that (3) implies the coefficient of V_1_ in formula (*) in Table 2 is positive, so that point A2 yields higher ATP production compared to point B.

**Table 2:** Critical points for linear programming model with limited substrate. Points A2 and A4 are feasible only if the corresponding values shown for *v*_3_ are non-negative and those for *v*_2_ are positive or negative, respectively.

Point	Binding Constraints	Values			
		*v*_1_	*v*_2_	*v*_3_	ATP value *ϕ*
O	(e)(f)	0	0	0	0
A	(c)(f)	$\frac{\Gamma }{\left(\alpha_{1}+\alpha_{2}\right)}$	$\frac{\Gamma }{\left(\alpha_{1}+\alpha_{2}\right)}$	0	$\frac{m_{1}\Gamma }{\left(\alpha_{1}+\alpha_{2}\right)}$
B	(c) & *v*_2_ ≥ 0	$\frac{\Gamma }{\left(\alpha_{1}+\alpha_{3}\right)}$	0	$\frac{\Gamma }{\left(\alpha_{1}+\alpha_{3}\right)}$	$\frac{(m_{1}+m_{3})\Gamma }{\left(\alpha_{1}+\alpha_{3}\right)}$
C	(d)(e)	0	$-\frac{\Gamma }{\left({\hat{\alpha }}_{2}+\alpha_{3}\right)}$	$\frac{\Gamma }{\left({\hat{\alpha }}_{2}+\alpha_{3}\right)}$	$\frac{m_{3}\Gamma }{\left({\hat{\alpha }}_{2}+\alpha_{3}\right)}$
A1	(f)(g)	V_1_	V_1_	0	*m*_1_V_1_
A2	(c)(g)	V_1_	$\frac{(\alpha_{1}+\alpha_{3})V_{1}-\Gamma }{\left(\alpha_{3}-\alpha_{2}\right)}$	$\frac{\Gamma -(\alpha_{1}+\alpha_{2})V_{1}}{\left(\alpha_{3}-\alpha_{2}\right)}$	$\frac{m_{3}\Gamma }{\alpha_{3}-\alpha_{2}}$ (*) $+\;{\text{V}}_{1}\left(m_{1}-m_{3}\frac{\alpha_{1}+\alpha_{2}}{\alpha_{3}-\alpha_{2}}\right)$
A3	(g) & *v*_2_ ≥ 0	V_1_	0	V_1_	(*m*_1_ + *m*_3_)V_1_
A4	(d)(g)	V_1_	$\frac{(\alpha_{1}+\alpha_{3})V_{1}-\Gamma }{\left(\alpha_{3}+{\hat{\alpha }}_{2}\right)}$	$\frac{\Gamma +({\hat{\alpha }}_{2}-\alpha_{1})V_{1}}{\left(\alpha_{3}+{\hat{\alpha }}_{2}\right)}$	$\frac{m_{3}\Gamma }{{\hat{\alpha }}_{2}+\alpha_{3}}$ $+\;{\text{V}}_{1}\left(m_{1}+m_{3}\frac{{\hat{\alpha }}_{2}-\alpha_{1}}{{\hat{\alpha }}_{2}+\alpha_{3}}\right)$

If the substrate limit is tightened to $V_{1}=\frac{\Gamma }{\alpha_{1}+\alpha_{3}}=3.92$, the ATP production is maximized by using pure respiration, marked point A2 = B in Fig 7. There are enough internal resources to process the small amount of substrate using the most costly pathway, yielding the maximum possible amount of ATP.

If the substrate limit is reduced further to $2=V_{1}<\frac{\Gamma }{\alpha_{1}+\alpha_{3}}$, then the optimal operating point is point A3 in Fig 8. The organism tends to purely use the costly respiration for metabolizing the scarce substrate to maximize the ATP production when it is not allowed to use the reversed fermentation. The organism has unused internal capacity and hence can process all the available substrate using pure respiration (point A3). If reverse fermentation is feasible, then it has the internal capacity to take advantage of reverse fermentation in addition to respiration to achieve a higher ATP production rate (point A4 in Fig 8).

We now discuss the case in which the respiration is low cost, that is *α*_3_ is small enough so (3) does not hold. We illustrate the situation using the same numerical values as before, except *α*_3_ = 10 [24]. As before, if $V_{1}\geq \frac{\Gamma }{\alpha_{1}+\alpha_{2}}=100$, the system behaves as if the substrate is unlimited, with the optimal operating point at point B in Fig 2. If we impose modest limits on the uptake of P_1_ (i.e., $18.18=\frac{\Gamma }{\alpha_{1}+\alpha_{3}}<V_{1}<\frac{\Gamma }{\alpha_{1}+\alpha_{2}}=100$), the optimal operating point is still pure respiration at point B in Fig 4, illustrated using V_1_ = 25. Here ATP production is still maximized by using pure respiration (point B). The organism tends to use respiration as the low cost but efficient mechanism, achieving a moderate amount of ATP production, without using all the available substrate. The coefficient of V_1_ in formula (*) in Table 2 is negative, indicating that vertex A2 corresponds to a lower ATP production rate compared to vertex B.

If we impose a tighter limit on the uptake of P_1_ (e.g., $V_{1}=3.92<\frac{\Gamma }{\alpha_{1}+\alpha_{3}}=18.18$), the optimal solution consists of a mix of respiration and reversed fermentation if the fermentation is reversible (point A4 in Fig 5), but it consists only of pure respiration if the fermentation is irreversible (point A3 in Fig 5).

### Discussion

In our model, the optimal energetic metabolic regimes in a minimal model describing respiration and fermentation can be predicted by the optimality criteria of maximizing ATP production rate. The optimization problems are linear with respect to the fluxes because ATP production rates are linear functions of fluxes and the side constraints are also linear. The possible non-linear underlying rate laws were analyzed by [8, 9]. The new aspect here is that fermentation is allowed to be reversible. Our analysis reveals that the Warburg effect allows the cell to invest into the oxidation of lactate and further respiration to produce more ATP. It is also of interest to analyze the relationship to the concept of elementary flux vectors [25].

It has been questioned whether cancer cells always show an increased ATP production and how they can make it [26]. We should note that the coefficients in 1(*c*), 1(*d*) and the availability of the substrates can be changed upon tumorigenesis. By our assumption, a cell can partially compensate for limited substrate availability by shifting to glycolysis or a “reversed” fermentation.

**Fig 9:**
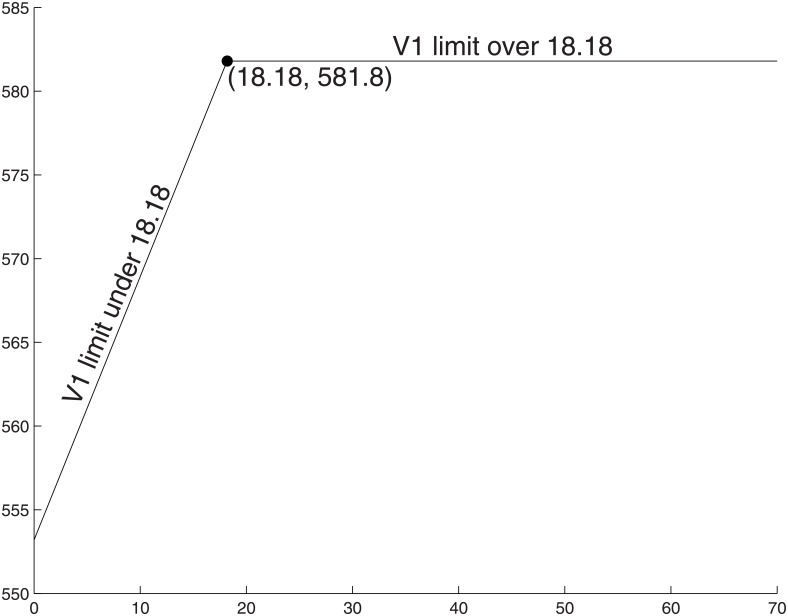
ATP production vs. substrate limits with low cost respiration. *α*_1_ = 1, *α*_2_ = 1, *α*_3_ = 10, Γ = 200, ${\hat{\alpha }}_{2}=1$ and *v*_1_ is capped by a value within the range [0, +∞). When the uptake of the substrate has a limit greater than 18.18, it will not affect the ATP production and it remains as 581.8. As shrinking the constraint, the ATP production rate is linearly decreased until 545.4 when there is no net external substrate provided. The turning point is at (18.18, 581.8).

We show in Fig 9 how the production of ATP varies with availability of glucose substrate when respiration is low-cost. When glucose availability is large (V_1_ ≥ Γ/(*α*_1_ + *α*_3_)), the ATP production is maximized out using pure respiration, being limited by the internal resource capacity of the cell (Fig 9 segment Q). When the glucose availability drops below the critical value (Γ/(*α*_1_ + *α*_3_)), the cells begin to make use of the energy from oxidation of lactate leading to more pyruvate, which facilitates the respiration pathway producing more ATP. The ATP production decreases linearly until a limit value, as illustrated in Fig 9 segment P. We see that even when no glucose is present at all, this system can still achieve an ATP production that is not much less than the maximum possible rate (545.4 moles or 93.75% = *m*_3_/(*m*_1_ + *m*_3_) of the maximum ATP production rate in our numerical example).

**Fig 10:**
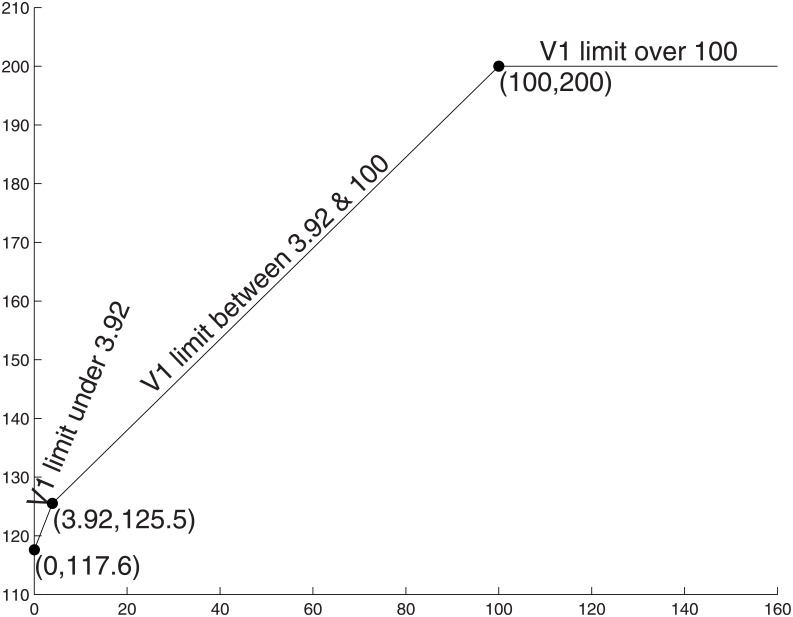
ATP production vs. substrate limits with high cost respiration. *α*_1_ = 1, *α*_2_ = 1, *α*_3_ = 50, Γ = 200, ${\hat{\alpha }}_{2}=1$ and *v*_1_ is capped by a value within the range [0, +∞). When the uptake of the substrate has limit greater than 100, it will not affect the ATP production and it remains as 200. As shrinking the constraint, the ATP production is linearly decreased until the limit reaches 3.92 in which *v*_2_ = 0, then the production is linearly decreased with a sharper speed until 117.6 when there is no net external substrate provided. The turning points are at (100, 200) and (3.92, 125.5).

In Fig 10 we show how the production of ATP varies when respiration carries a high cost. The ATP production varies while the substrate limits gradually shrinks to 0. With an available amount greater than 100, ATP production remains flat at 200 (Fig 10 segment X), using pure fermentation. When the glucose availability falls below the break-even point (Γ/(*α*_1_ + *α*_2_)), the cell begins to make use of the energy from a mix of fermentation and respiration, but ATP production gradually decreases (Fig 10 segment Y).

The cell tends to use more fermentation because of the expensive cost of respiration but will gradually balance the difference as the limit becomes tighter. When glucose availability is decreased to the second cross-over point (Γ/(*α*_1_ + *α*_3_)), the cell is using pure respiration to produce less ATP (125.5 moles in our numerical example). When glucose availability falls below this second cross-over point, our model predicts that the cell resorts to consuming lactate to keep the respiration process at full capacity. This is indeed observed in neurons [27, 28] and in some muscle cells [29] in addition to glucose. The brain does not need to take up lactate unless the body is hypoglycaemic. Another example is the above mentioned cross-feeding species of *E. coli* where the first strain has an advantage in growth rate on glucose. The second strain is reliant on the products excreted by the first one, because it would have no chance surviving when only relying on glucose. The same occurs for muscle cells if one group of muscle cells is under high physical stress a different group of muscles takes up lactate instead of glucose. This could occur for several reasons, first to keep their energy-level up. Second because they should not take away the valuable resource from the muscles that have to deal with the workload. The third reason could be that they clear the tissue from the lactate, so that an acidification can be prevented.

Extending previous models of the Warburg effect [4–6, 18], the present model can thus explain the respiration of fermentation products based on an optimality principle (Fig 10 segment Z). When glucose is entirely absent, but lactate is fully available, the cell is still able to produce ATP at a rate comparable to the maximum rate when glucose is unlimited (in our example 117.6 moles of ATP, $58\%=\frac{m_{3}}{{\hat{\alpha }}_{2}+\alpha_{3}}\times \frac{\alpha_{1}+\alpha_{2}}{m_{1}}$ of the maximum possible production).

The approach of maximizing the ATP synthesis flux is based on the assumption that cells generating ATP as fast as possible should have a selective advantage. This is likely to be particularly relevant for microorganisms, which can out-compete other species or strains when growing fast and for rapidly proliferating cells in multicellular organisms. It is also useful for predicting the behavior of organisms who co-live within a certain area.

## Conclusions

We use a simple linear programming model to illustrate how a simple metabolic system can use a mix of processes to achieve the optimal energy production under a variety of conditions. Under conditions in which respiration is low cost, the optimal solution for maximizing the ATP production is pure respiration. But if the input substrate is tightly limited, the favored solution will be a mixture of respiration and reversed fermentation if it is allowed. Under conditions in which respiration is costly, the optimal solution will be pure fermentation when the substrate is unlimited. But if the input resource is limited, the favored solution will be a mixture of respiration and fermentation or pure respiration or a combination of reversed fermentation and respiration under very severe limits. A somewhat more complicated model would be required if there were limits on the substrate consumed by reverse fermentation, or if there were alternate objectives beyond ATP, such as NADH.

## Supporting information

S1 Parameter Values Table of parameter values for Figs 2–8. (CSV)
